# The Effects of Food Taxes and Subsidies on Promoting Healthier Diets in Iranian Households

**DOI:** 10.3389/fnut.2022.917932

**Published:** 2022-07-13

**Authors:** Amin Mokari-Yamchi, Nasrin Omidvar, Morteza Tahamipour Zarandi, Hassan Eini-Zinab

**Affiliations:** ^1^Department of Community Nutrition, Faculty of Nutrition Sciences and Food Technology, National Nutrition and Food Technology Research Institute, Shahid Beheshti University of Medical Sciences, Tehran, Iran; ^2^Department of Economics, Faculty of Economics and Political Science, Shahid Beheshti University, Tehran, Iran

**Keywords:** fiscal policy, taxes, almost ideal demand system, Iran, diet

## Abstract

**Background and Aim:**

Price, as a key driver of food purchasing, has an important role in determining the consumer demand. This study is aimed to estimate the effect of food taxes and subsidies on purchasing patterns of Iranian households (HHs).

**Methods:**

This study was performed in two phases. In phase one, a two-round Delphi study was conducted to determine and prioritize food-related fiscal policies; and in the second phase, using the Iranian Household Income and Expenditure Survey (HIES), we estimated an almost ideal demand system (AIDS) and simulated changes in purchases, nutrient intake, and consumer welfare under six different policy scenarios: (1) 20% subsidy on vegetables, (2) 20% subsidy on fruits, (3) 30% subsidy on legumes, (4) 25% tax on sugar and sweets, (5) 30% tax on sweetened beverages, and (6) 30% tax on hydrogenated oil and animal fats.

**Results:**

The highest calorie reduction was detected in sugar and sweets tax, which has resulted in 949.67, 971.68, and 1,148.03 kilocalories decrease in energy intake per Adult Male Equivalent (AME) in all HHs, low-income HHs, and high-income HHs, respectively. In terms of welfare changes, high-income HHs will experience a lower change in welfare (−0.81 to 0.11%) relative to their income when compared with low-income HHs (−0.88 to 0.28%) due to fiscal policies.

**Conclusion:**

Fiscal policies in Iran can be a potential way to improve dietary choices. The findings provide essential information for decision makers for the implementation of food-related fiscal policies.

## Background

Non-communicable diseases (NCDs) have been the most important global health problem over the last decades and are responsible for almost 40 million deaths each year (equivalent to 70% of all deaths, globally). The relationship between NCDs and unhealthy foods and drinks’ consumption is well known. It has been shown that the dietary factors contribute to almost 10% of the global burden of diseases (1). In response to the growing evidence on the causal relationship between an unhealthy diet and increased NCDs risk, food and nutrition experts suggest different strategies that can contribute toward a healthy community, such as promotion of nutrition literacy, controlling food products advertising, food labeling, and food-related fiscal policies through taxation or subsidies (2, 3).

Since the early 1980s, several countries have implemented taxes on unhealthy foods and beverages, for revenue purposes and to reduce demand for their consumption (4). Fiscal policies have also been recommended by the World Health Organization (WHO) as a proper way to discourage the consumption of unhealthy foods and beverages (5). Price, as a key driver of food purchasing, has an important role in determining the consumer demand. Previous studies in real-world environments indicated that people tend to reduce their consumption of unhealthy foods as prices increase (6). To guide policy-making, research has an important role in estimating the likely impact of such fiscal policies on changes in household (HH) diet. A large number of recent studies have concentrated on estimating the impact of price changes on the demand for certain food categories, such as sugar-sweetened beverages (SSBs) (7, 8), saturated fats (9), and fruit and vegetables (10, 11).

One criticism commonly stated about food-related fiscal policies is that HHs in low- and middle-income countries would pay a greater percentage of their income on food and evidence from high-income countries may not be directly applicable to middle- and low-income countries (12). Alagiyawanna et al. conducted a systematic review to study the different outcomes of fiscal policies in countries of different income classifications. The results of their study supported previous findings that fiscal policies can have an impact on healthy food consumption; it also highlights the lack of enough evidence in low- and middle-income countries in this regard (13).

In Iran, since 2012, the annual average rate of food inflation has been over 29% (14). Previous studies conducted in the country have mainly focused on price increases and their impact on changes in HH welfare (15–17). To the best of our knowledge, health-related targeted food taxes and subsidies have not been studied so far. Thus, the aim of this study was to estimate the effects of targeted food taxes and subsidies on the food purchasing patterns of Iranian HHs.

## Materials and Methods

This study was performed in two phases. In phase one, policy options for food taxation or subsidizing were prioritized; and in the second phase, the effects of pricing policy on different income groups were evaluated using the existing data.

### Phase 1: Food Tax and Subsidy Scenarios

This qualitative study was conducted from May to November 2021 using the Delphi approach. A Delphi study was performed on a panel that consisted of Iranian researchers who had published on food-related fiscal policies, health managers, and other experts in health and nutrition policy-making. Twenty-seven participants took part in this two-round Delphi study. The general characteristics of the participants are presented in the Supplementary Table 1. In the first round, the main question asked through the Delphi phases was “considering health issues, what food items or groups are appropriate and have the highest priority to be taxed or subsidized in Iran?”

As an indicator of consensus, the quantitative summary [mean, standard deviation (SD), median, and mode] of responses was calculated. A second questionnaire was created with the policy options proposed by the participants. Following that, the questionnaire was sent to all participants in case they wanted to change their opinion regarding the quantitative information of each policy option. Eventually, according to the consensus (mode) of responses, three tax scenarios and three subsidy scenarios were selected, which were as follows: (1) 20% subsidy on vegetables, (2) 20% subsidy on fruits, (3) 30% subsidy on legumes, (4) 25% tax on sugar and sweets, (5) 30% tax on SSBs, and (6) 30% tax on hydrogenated oil and animal fats. In the second round, the panel was asked to rate policy options with regard to six factors (impact of policy option on health, chance of stability, implementation feasibility, implementation costs, acceptance by authorities, and acceptance by authorities society) on a 5-point Likert scale (1 = very low, 2 = low, 3 = moderate, 4 = high, and 5 = very high). Data were analyzed using basic descriptive statistical tests and expressed as mean and SD. In addition, the highest mean indicated an option has high priority.

### Phase 2: Evaluating Food Price Policy Effect

In this phase of the study, data from Iran’s Households Income and Expenditure Survey (HIES) that is being carried out annually by the Statistical Centre of Iran were utilized. HIES provides information on an HH’s living conditions and income/expenditure patterns. In the present study, we used data from 1990 to 2021. The sample size in this study at the national level was 927,680 HHs. In order to examine the pricing policy effects on different income groups, we created three sub-samples using the tertile of the HH income distribution, where lower tertile refers to HHs with lower income and likewise for higher tertile.

### Demand Model

The Almost Ideal Demand System (AIDS) of Deaton and Muellbauer (18) by the method of seemingly unrelated regressions (SURs) was applied to estimate demand parameters for food groups. The AIDS model allows us to predict the potential demand response to changes in prices. All estimations were carried out using Eviews Software version 10 and Microsoft Excel 2010. The model is specified as follows:


$$w_{i}=\alpha_{i}+\Sigma \,\gamma_{i\,j}\,l\,n\,p_{j}+\beta_{i}\,\ln\left(M\,t_{i}/P\right)+t_{i}\,d$$

Where *w*_*i*_is the food or beverage expenditure share for food or beverage group i; *p*_*j*_is the unit value for food or beverage j; *Mt*_*i*_is real HH income; d is the family size, and P is the stone price index, defined as follows:


$$l\,n\,P=\sum_{i=1}^{n}w_{i}+\ln p_{i}$$

Marshallian and Hicksian price elasticities of the demand for the food groups were calculated using the following equations:


$$\varepsilon =-\delta_{i\,j}+\frac{\gamma_{i\,j}}{w_{i}}-\beta_{i}\,\left(\frac{w\,j}{w_{i}}\right)$$

$$\varepsilon =-\delta_{i\,j}+\frac{\gamma_{i\,j}}{w_{i}}+w_{j}$$

Where ε is the price elasticity of the food or beverage category, δ equals 1 if it is own-price elasticity and 0 if cross-price elasticity, *w* is the mean expenditure share of food or beverage, β_*i*_ is the estimated parameter of the log real income, and γ_*ij*_ is the estimated parameter associated to the unit value of the food or beverage category. Additionally, the welfare change to consumers is estimated using the compensating variation (CV) measure, reflecting the income an HH needs to receive in order to return to the original utility level after a price change. Hicksian elasticities were used to calculate the CV as an established approach in the literature (19):


$$△\,l\,n\,c=\Sigma_{i}\,w_{i}\,△\,l\,n\,p_{i}+1/2\,\Sigma_{i}\,\Sigma_{j}\,w_{i}\,\varepsilon_{i\,j}\,△\,l\,n\,p_{i}\,△\,l\,n\,p_{j}$$

### Change in Dietary Pattern

The 2020–2021 HIES data were applied to estimate the impact of food-related fiscal policies on the percent changes in HH purchases and also the amount of calorie and nutrient intake. The total sample size was 37,557 HHs. We determined the change in monthly intake of foods (e.g., amount of fruits and vegetables) and nutrients (e.g., carbohydrates and saturated fat) for each tax and subsidy scenario, using price elasticity data. Since data on the HH food basket were collected for the HHs as the sampling units, they were converted into individual amounts. The estimates were calculated per Adult Male Equivalent (AME) unit per month. AME is the ratio of the energy requirement of an HH member of a particular age and gender to the energy requirement of an adult male aged 18–30 years with moderate physical activity as recommended by the Food and Agriculture Organization (FAO) and WHO (20). In the present study, the total AME of the HH was calculated; then, the amount of each food item was divided by the total AME of the HH. Since purchased food is partly wasted, the real amount of the consumed foods was estimated based on the FAO-recommended waste percentage for each food group in the consumption phase (21). Food analyses were performed using the Nutritionist IV software, and the AME of the energy and nutrient intake were calculated manually.

## Results

Table 1 shows the policy options prioritized by the expert panel. The panel suggested that subsidizing vegetables had the greatest impact on health (mean score = 4.18) and acceptance by society (3.85), with the greatest implementation cost (3.88). In regards to “implementation feasibility” and “acceptance by the authorities,” the tax on sweetened beverages had the highest agreement. Furthermore, taxation of hydrogenated oil and animal fats was considered to have the highest chance of stability (3.37).

**TABLE 1:** The mean (SD) scores of the prioritization criteria for each policy.

		Impact on health	Chance of stability	Implementation feasibility	Implementation costs	Acceptance by the authority	Acceptance by the society
Subsidy scenarios	20% on vegetables	4.18 (0.78)	2.4 (0.84)	2.37 (0.83)	3.88 (1.01)	1.92 (0.78)	3.85 (0.98)
	20% on fruits	4 (0.73)	2.62 (0.88)	2.55 (1)	3.59 (1)	2.07 (0.67)	3.81 (0.78)
	30% on legumes	3.88 (0.75)	2.52 (1.04)	2.25 (0.81)	3.74 (0.94)	2.18 (0.87)	3.74 (1.2)
Tax scenarios	25% on sugar	3.88 (0.84)	2.88 (0.84)	3.55 (1.08)	2.77 (0.75)	3.18 (1.07)	2.66 (1)
	30% on sweetened beverages	3.66 (0.91)	2.62 (1)	3.96 (1.05)	2.4 (0.79)	3.33 (1.07)	2.81 (0.92)
	30% on hydrogenated oil and animal fats	3.7 (0.86)	3.37 (1.04)	3.55 (1.15)	2.77 (1.08)	2.96 (1.02)	2.25 (1.1)

*A 5-point Likert scale (1 = very low to 5 = very high) was used for scoring. SD, standard deviation.*

Table 2 presents the descriptive statistics for the all HHs, low-income HHs, and high-income HHs defined as the first and third tertiles of the HH annual income distribution, respectively. The average age of the HH heads age was 51.87 years, while 85% of the HHs were male-headed, and the average HH size was about 3.43 people. Moreover, it is noted that low-income HHs had a mean income of 24,129.42 thousand Rials per month, whereas the mean income of high-income HHs was 68,802.3 thousand Rials/month.

**TABLE 2:** Household descriptive statistics.

	All HHs	Low income HHs	High income HHs
Household head’s age	51.87 (15.28)	54.4 (16.88)	47.73 (13.35)
Household head’s education (illiterate %)	20.35	32	9.37
Female household head (%)	14.7	21	4.2
Household size	3.43 (1.44)	3.29 (0.51)	3.95 (0.81)
Households income (thousand rials)	39710.16 (48835.92)	24129.42 (17417.82)	68802.3 (57439.2)

*HHs, households. Standard deviations (SDs) are in parentheses.*

Table 3 provides descriptive statistics on the 11 food categories for both total HHs and sub-samples by HH income. We report unit value (in thousand Rials), purchase quantity [in kilograms or liters (kg or L)], and budget share (% of income). Cereals in all groups have the highest budget share and quantity. In addition, red meat and vegetables have the highest and lowest unit prices, respectively. It has been observed that high-income HHs purchase more on every food group (except hydrogenated oils and animal fats), pay higher unit values, and spend a smaller share of their budget than low-income HHs.

**TABLE 3:** Food group statistics by household income group.

	Unit value (thousand rials)	Share (%)	Quantity (kg/liter)
**All HHs**			
Cereals	59.59	7.01	46.95
Un-hydrogenated vegetable oils	128.69	0.69	2.13
Hydrogenated oils and animal fats	209.99	0.66	1.27
Red meat	746.76	3.41	1.82
White meat and eggs	209.91	3.85	7.7
Dairies	83.42	2.14	10.24
Sugar and sweets	111.72	0.99	3.56
Fruits	94.28	3.32	13.95
Vegetables	48.66	2.9	23.78
Legumes	228.81	0.9	1.57
Sweetened beverages	50	0.41	3.3
**Low income HHs (tertile 1)**			
Cereals	46.2	9.99	52.04
Un hydrogenated vegetable oils	122.64	0.97	1.92
Hydrogenated oils and animal fats	166.74	1.03	1.5
Red meat	658.98	3.52	1.29
White meat and eggs	189	5.44	7.23
Dairies	76.86	2.86	9.05
Sugar and sweets	107.1	1.61	3.63
Fruits	78.96	3.63	11.55
Vegetables	45.36	4.17	21.98
Legumes	214.2	1.25	1.41
Sweetened beverages	48.76	0.57	2.78
**High income HHs (tertile 3)**			
Cereals	69.3	5.31	58.13
Un hydrogenated vegetable oils	133.56	0.5	2.87
Hydrogenated oils and animal fats	262.06	0.49	1.46
Red meat	769.44	3.21	3.19
White meat and eggs	231	2.96	10.3
Dairies	88.62	1.7	14.69
Sugar and sweets	116.34	0.67	4.42
Fruits	108.36	2.99	20.74
Vegetables	51.24	2.19	32.29
Legumes	240.24	0.68	2.16
Sweetened beverages	51.66	0.34	5.24

*HHs: households. Unit values are expressed in Thousand Rials. Quantities are measured in kg or L depending on each food group. Shares represent the fraction of the total income (%).*

Estimated Marshallian own- and cross-price elasticities are presented in the Supplementary Tables 2–4. Based on the Marshallian elasticities, the variations in purchases due to each fiscal policy for each food group were computed. The percent changes for all food groups are reported in Table 4. According to the table, legumes are more price elastic as compared to other targeted food groups in all groups (−1, −0.97, and −1.08 for all HHs, low-income HHs, and high-income HHs, respectively). The strongest demand responses were observed in policy 3, which would lead to an overall purchase increase of 30.24, 29.37, and 32.47% of legumes in all, low-income HHs, and high-income HHs, respectively. Whereas the weakest responses were detected in policy 1, that as a result, the overall purchase of vegetables would increase by 7.49, 5.5, and 9.63%, respectively, for the all, low-income HHs, and high-income HHs.

**TABLE 4:** Percentage change in household purchases due to fiscal policies.

	Policy 1	Policy 2	Policy 3	Policy 4	Policy 5	Policy 6
**All HHs**						
Cereals	0.92	0.46	−0.71	0.55	−0.35	0.52
Un-hydrogenated	−1.81	−1.06	3.5	1.58	0.97	1.56
vegetable oils						
Hydrogenated oils	1.14	1.12	1.07	−1	−0.78	−27
and animal fats						
Red meat	−0.46	−0.41	2.49	0.43	0.46	−2.92
White meat and eggs	2.28	−0.46	2.12	−1.51	2.34	0.62
Dairies	−0.67	−2.46	0.26	1.32	1.88	0.005
Sugar and sweets	−2.11	−2.59	−0.12	−18.7	−3.52	−2.8
Fruits	−0.95	13.07	0.6	−0.86	1.39	−0.79
Vegetables	7.49	6.45	−1.37	−3.14	1.47	−1.2
Legumes	1.4	−3.25	30.24	−1.66	1.89	0.8
sweetened beverages	2.75	2.87	0.42	1.18	−13.8	−2.44
**Low income HHs (tertile 1)**						
Cereals	1.31	0.72	−1.22	0.77	−0.48	1.19
Un hydrogenated	−1.03	−0.96	−0.82	−1.15	2.78	1.66
vegetable oils						
Hydrogenated oils	2.11	1.78	4.45	2.04	−2.73	−14.69
and animal fats						
Red meat	2.46	2.83	1.23	−0.93	2.66	−2.31
White meat and eggs	1.77	0.47	0.92	0.96	−0.97	−0.39
Dairies	−2.56	−2.54	1.8	2.12	−1.06	−0.79
Sugar and sweets	−1.74	0.83	0.18	−23.78	−1.49	0.38
Fruits	3.97	13.43	1.88	−1.96	−1.52	−2.31
Vegetables	5.5	2.81	2.97	−0.85	0.33	−1.31
Legumes	3.85	4.97	29.37	−0.33	1.53	−3.84
Sweetened beverages	0.97	1.5	−4.48	3.54	−17.76	−1.48
**High income HHs (tertile 3)**						
Cereals	0.1	−0.59	0.13	−0.01	0.33	0.9
Un hydrogenated	−0.53	1.52	1.53	2.95	−4.5	5.61
vegetable oils						
Hydrogenated oils	2.07	2.32	2.81	2.1	−0.87	−31.87
and animal fats						
Red meat	−1.97	0.16	−2.25	−0.44	−1.79	−2.81
White meat and eggs	0.14	0.94	−2.45	0.21	0.22	−1.51
Dairies	2.63	0.02	3.35	−1.64	−1.97	1.4
Sugar and sweets	1.83	−0.62	1.78	−21.6	−4.89	0.56
Fruits	−3.31	20.62	−0.06	−0.71	−3.22	−2.73
Vegetables	9.63	−0.47	−2.19	−0.41	0.51	0.76
Legumes	2.75	−0.01	32.47	3.22	−2.9	5.37
Sweetened beverages	1.39	1.67	−0.61	−2.03	−13.2	−2.7

*HHs: households. Each policy is defined in Table 1. Underlined estimates differ significantly from 0 at the 5% significance level.*

Table 5 shows the percentage changes in HH purchases converted to calorie and nutrient intake per AME per month. All policies affect the amount of nutrients intake. For example, all subsidy policies increased energy and dietary fiber intake in all groups. The sweetened beverage tax produced 144.23, 450.04, and 977.94 kilocalories decrease in energy intake per AME in all, low-income HHs, and high-income HHs, respectively. The sugar and sweets tax gave a notable reduction in sugar intake (235.13, 292.35, and 301.17 g per AME per month in all, low-income HHs, and high-income HHs, respectively; Table 5).

**TABLE 5:** Change in nutrient intake per AME per month.

	Policy 1	Policy 2	Policy 3	Policy 4	Policy 5	Policy 6
**All HHs**						
Kilocalories (kcal)	538.48	509.21	685.03	−949.67	−144.23	−933.12
Carbohydrate (gr)	113.34	136.75	9.34	−248.42	−77.33	−5.45
Protein (gr)	30.48	13.49	43.18	−6.75	10.25	6.12
Fat (gr)	−2.18	2.47	53.84	0.48	9.75	−115.15
Saturated Fat (gr)	1.04	0.41	8.06	−1.14	1.72	−21.86
Sugar (gr)	−14.92	27.77	6.5	−235.13	−39.89	−39.94
Vitamin C (mg)	60.31	224.39	2.8	−39.45	22.05	−22.85
Dietary Fiber (gr)	11.36	15.02	16.36	−3.38	2.49	0.36
**Low income HHs (tertile 1)**						
Kilocalories (kcal)	935.29	1348.41	276.26	−971.68	−450.04	−245.55
Carbohydrate (gr)	177.11	287.33	−30.18	−273.04	−105.11	91.71
Protein (gr)	38.91	41.31	26.95	9.36	−8.63	4.77
Fat (gr)	13.65	13.48	32.11	−0.89	2.49	−72.06
Saturated Fat (gr)	3.36	2.86	6.5	0.71	−0.9	−14.22
Sugar (gr)	−1.1	55.68	21.13	−292.35	−29.03	−7.61
Vitamin C (mg)	89.61	165.94	49.11	−25.16	−22.98	−39.43
Dietary Fiber (gr)	14.41	18.68	16.49	0.55	−1.55	−0.9
**High income HHs (tertile 3)**						
Kilocalories (kcal)	665.89	795.11	959.63	−1148.03	−915.4	−420.48
Carbohydrate (gr)	126.95	128.06	108.13	−361.96	−105.36	110.97
Protein (gr)	15.1	10.94	34.28	−17.3	−2.5	9.98
Fat (gr)	5.66	40.3	37.31	33.34	−52.31	−106.91
Saturated Fat (gr)	1.07	5.67	4.31	4.24	−6.21	−24.21
Sugar (gr)	35.28	125.7	35.23	−301.17	−92.81	−1.35
Vitamin C (mg)	54.07	375.77	−15.8	−18.6	−63.12	−39.1
Dietary Fiber (gr)	12.67	16.56	22.34	1.15	−3.25	6.3

*HHs: households. Each policy is defined in Table 1. Underlined estimates differ significantly from 0 at the 5% significance level.*

A comparison of the welfare change (measured by CV) is presented in Table 6 in both thousand Rials and as a percentage of HH income. The welfare benefits of subsidy policies (policy 1–3) for households were 254.52, 308.28, and 140.7 thousand Rials per month, respectively (0.64, 0.77, and 0.35% of income). In addition, the welfare costs of tax policies (policy 4–6) for HHs were 66.78, 43.26, and 60.9 thousand Rials per month, respectively (0.16, 0.1, and 0.15% of income). There is heterogeneity in the welfare changes of policies between low- and high-income HHs. In terms of welfare changes, high-income HHs will experience a lower welfare cost due to tax policies relative to their income (0.09–0.11% vs. 0.14–0.28%). In addition, in subsidy policies, low-income HHs receive a larger welfare benefit as a share of their income as compared to high-income HHs (−0.46 to −0.88% vs. −0.28% to −0.81%; Table 6).

**TABLE 6:** Households welfare change (compensated variation as % of income and Thousand Rials per month) due to price changes.

		All HHs		Low income HHs		High income HHs	
		% of income	Thousand Rials	% of income	Thousand Rials	% of income	Thousand Rials
Subsidy scenarios	20% on vegetables	−0.64	−254.52	−0.88	−214.2	−0.5	−347.76
	20% on fruits	−0.77	−308.28	−0.85	−206.64	−0.81	−561.54
	30% on legumes	−0.35	−140.7	−0.46	−111.72	−0.28	−196.14
Tax scenarios	25% on sugar and sweets	0.16	66.78	0.28	68.46	0.11	78.96
	30% on sweetened beverages	0.1	43.26	0.14	34.86	0.09	64.26
	30% on hydrogenated oil and animal fats	0.15	60.9	0.25	62.58	0.11	79.8

*HHs: households. Positive values denote welfare losses, whereas negative values denote welfare gains. Underlined estimates differ significantly from 0 at the 5% significance level.*

## Discussion

This study modeled the suggested food-related fiscal policies by experts on food demand in Iran. According to the experts, subsidy policies would have a greater impact on health, but a lower chance of stability, whereas these policies would have a higher possibility of acceptance by the society. Since subsidies impose a lot of costs on the government, it is expected that they have a lower chance of acceptance by authorities. A 2020 study by Blakely et al. raised concern that there are multiple considerations regarding fiscal policies, such as political and social acceptability and costs of any tax or subsidy (22). To the best of our knowledge, this is the first study that has been subjected to such a process to determine the most appropriate food-related fiscal policy.

We estimated a set of elasticities to simulate percent changes, nutrient intake, and welfare losses (or gains) under different policy scenarios. Our estimations are based on the all HHs, as well as HHs of first and third income tertiles, recognizing that HHs in different tertiles may have different preferences. The low-income HHs in our sample are less price responsive (except for sugar and sweets and sweetened beverages) than the high-income HHs, and so the changes in their demands were less than those of the high-income HHs. This may be because low-income families devote a large percentage of their budget to these food categories (35.04%) rather than high-income families (21.04%). As a comparison, Caro et al. found that low-income HHs report a lower own-price elasticity for the majority of food categories except for sweets and snacks (10). Moreover, Caro et al. in another study reported that SSBs, sweets and desserts, and salty snacks are more elastic for low-income HHs when compared with high-income HHs (23).

For understanding the health implications of these policies, we converted the percent changes in HH purchases into calorie and nutrient intakes per AME per month. Consistent with the Cobiac et al’s study (24), our results showed that tax policies decreased calorie intake and subsidy policies increased it. Among all the tax policies, sugar and sweets taxation was the most likely policy to lower calorie intake in the all HHs and among low- and high-income HHs. As expected, subsidy on fruits, vegetables, and legumes increased dietary fiber intake. The hydrogenated oil and animal fat tax reduced saturated fats, and the sugar and sweetened beverages tax reduced sugar intake. In addition, we found that some food taxes and subsidies could have unintended substitution effects, where, for example, a legumes subsidy might increase saturated fat consumption and limit health gains, or even cause harm. In previous simulation studies, it was shown that when food-related fiscal policies were implemented, some deleterious substitutions were expected, but the net health benefits were still high (22, 25).

In the present study, CV was used to estimate the costs of policies. As a result, the subsidy on the fruits was the most costly for the government (308.28 thousand Rials per HH as an indirect transfer to HHs). In absolute terms, we found that low-income HHs experience the largest relative welfare loss and gain (as a share of income) for tax and subsidy policies, respectively. The results of the current study confirm those of Zhen et al. (26) who found that the amount of compensation to erase direct welfare loss from increasing the price of sugar-sweetened beverages is much greater for low-income HHs in the United States. Evidence from Chile also indicates tax policy will result in a lower welfare cost for high-income HHs when compared with low-income HHs, relative to their average monthly income (10). Thus, welfare changes across socioeconomic subgroups depend not only on the price elasticity of demand but also on the initial level of consumption.

This study has a number of limitations. First, As noted in other studies (10, 23), we only examined the demand for foods and beverages and did not consider other substitutions and income effects beyond these purchases. Second, the data did not allow us to further separate food items, (e.g., whole grains from other cereals). Third, we examined the food-related fiscal policy effect on purchasing patterns and nutrient intake but did not consider the health impact of the policies. Fourth, we assumed that price changes would be fully passed onto the consumers, if it was not fully (but partially) passed onto them, the welfare changes from the policies would be undermined. The main strength of our study was using AME to calculate calorie and nutrient intake, as well as FAO estimates of waste percentages in order to be close to the real consumption rates of the individuals and to avoid possible underestimation and overestimation.

## Conclusion

As fiscal policies are increasingly gaining policy makers’ attention to promote healthier purchases, our estimates provide essential information for decision makers for the implementation of such policies. Our results indicated that the demand responses to the price policies that we have studied were strongest for legumes and weakest for vegetables in all, low-income HHs, and high-income HHs. Panelists also agreed that subsidies for vegetables would have the greatest impact on health and would have the greatest likelihood of being accepted by society, as well as being the most expensive to implement. Moreover, we found that low-income HHs experience the largest relative loss and gains in welfare (as a share of income) in response to taxes and subsidies, respectively.

## Data Availability Statement

The original contributions presented in this study are included in the article/Supplementary Material, further inquiries can be directed to the corresponding authors.

## Author Contributions

HE-Z: conceptualization, writing – review and editing, and supervision. MT: study design, guidance, and review of data analysis. AM-Y: writing – original draft preparation, methodology, software, and investigation. NO: conceptualization and writing – review and editing. All authors read and approved the final manuscript.

## Conflict of Interest

The authors declare that the research was conducted in the absence of any commercial or financial relationships that could be construed as a potential conflict of interest.

## Publisher’s Note

All claims expressed in this article are solely those of the authors and do not necessarily represent those of their affiliated organizations, or those of the publisher, the editors and the reviewers. Any product that may be evaluated in this article, or claim that may be made by its manufacturer, is not guaranteed or endorsed by the publisher.

## References

[1] GBD 2015 Risk Factors Collaborators. Global, regional, and national comparative risk assessment of 79 behavioural, environmental and occupational, and metabolic risks or clusters of risks, 1990–2015: a systematic analysis for the Global Burden of Disease Study 2015. Lancet. (2016) 388:1659–724. 10.1016/S0140-6736(16)31679-8 PMC5388856 27733284

[2] Vettori V Lorini C Milani C Bonaccorsi G. Towards the implementation of a conceptual framework of food and nutrition literacy: providing healthy eating for the population. *Int J Environ Res Public Health.* (2019) 16:5041. 10.3390/ijerph16245041 31835678 PMC6950737

[3] Buyuktuncer Z Ayaz A Dedebayraktar D Inan-Eroglu E Ellahi B Besler HT. Promoting a healthy diet in young adults: the role of nutrition labelling. *Nutrients.* (2018) 10:1335. 10.3390/nu10101335 30241289 PMC6213180

[4] Mytton OT Clarke D Rayner M. Taxing unhealthy food and drinks to improve health. *BMJ.* (2012) 344:e2931. 10.1136/bmj.e2931 22589522

[5] World Health Organization [WHO]. *Fiscal Policies for Diet and Prevention of Noncommunicable Diseases: Technical Meeting report; 5–6 May 2015.* Geneva: World Health Organization (2016).

[6] Epstein LH Jankowiak N Nederkoorn C Raynor HA French SA Finkelstein E. Experimental research on the relation between food price changes and food-purchasing patterns: a targeted review. *Am J Clin Nutr.* (2012) 95:789–809. 10.3945/ajcn.111.024380 22378726 PMC3302358

[7] Guerrero-López CM Unar-Munguía M Colchero MA. Price elasticity of the demand for soft drinks, other sugar-sweetened beverages and energy dense food in Chile. *BMC Public Health.* (2017) 17:180. 10.1186/s12889-017-4098-x 28183287 PMC5301435

[8] Schwendicke F Stolpe M. Taxing sugar-sweetened beverages: impact on overweight and obesity in Germany. *BMC Public Health.* (2017) 17:88. 10.1186/s12889-016-3938-4 28095809 PMC5240244

[9] Blakely T Nghiem N Genc M Mizdrak A Cobiac L Mhurchu CN Modelling the health impact of food taxes and subsidies with price elasticities: the case for additional scaling of food consumption using the total food expenditure elasticity. *PLoS One.* (2020) 15:e0230506. 10.1371/journal.pone.0230506 32214329 PMC7098589

[10] Caro JC Valizadeh P Correa A Silva A Ng SW. Combined fiscal policies to promote healthier diets: effects on purchases and consumer welfare. *PLoS One.* (2020) 15:e0226731. 10.1371/journal.pone.0226731 31940371 PMC6961856

[11] Broeks MJ Biesbroek S Over EA van Gils PF Toxopeus I Beukers MH A social cost-benefit analysis of meat taxation and a fruit and vegetables subsidy for a healthy and sustainable food consumption in the Netherlands. *BMC Public Health.* (2020) 20:643. 10.1186/s12889-020-08590-z 32389120 PMC7212616

[12] Andreoli V Bagliani M Corsi A Frontuto V. Drivers of protein consumption: a cross-country analysis. *Sustainability.* (2021) 13:7399. 10.3390/su13137399

[13] Alagiyawanna A Townsend N Mytton O Scarborough P Roberts N Rayner M. Studying the consumption and health outcomes of fiscal interventions (taxes and subsidies) on food and beverages in countries of different income classifications; a systematic review. *BMC Public Health.* (2015) 15:887. 10.1186/s12889-015-2201-8 26369695 PMC4570679

[14] Plan and Budget Organization. *Consumer Price Index.* Available online at: https://www.amar.org.ir/english/Statistics-by-Topic/Price-indices#2225496-releases (Accessed March 11, 2022).

[15] Layani G Bakhshoodeh M Aghabeygi M Kurstal Y Viaggi D. The impact of food price shocks on poverty and vulnerability of urban households in Iran. *Bio Based Appl Econ.* (2020) 9:109–25.

[16] Akbari A Ziaei MB Ghahremanzadeh M. Welfare impacts of soaring food prices on Iranian urban households: evidence from survey data. *Int J Bus Dev Stud.* (2013) 5:23–38.

[17] Ghahremanzadeh M Ziaei MB. Food price change and its welfare impact on Iranian households. *Int J Agric Manage Dev.* (2014) 4:313–23.

[18] Deaton A Muellbauer J. An almost ideal demand system. *Am Econ Rev.* (1980) 70:312–26.

[19] Tiezzi S. The welfare effects and the distributive impact of carbon taxation on Italian households. *Energy Policy.* (2005) 33:1597–612. 10.1016/j.enpol.2004.01.016

[20] Weisell R Dop MC. The adult male equivalent concept and its application to household consumption and expenditures surveys (HCES). *Food Nutr Bull.* (2012) 33:157–62. 10.1177/15648265120333S203 23193766

[21] Gustafsson J Cederberg C Sonesson U Emanuelsson A. *The Methodology of the FAO Study: Global Food Losses and Food Waste-Extent, Causes and Prevention – FAO, 2011.* Rome: SIK Institutet för livsmedel och bioteknik (2013).

[22] Blakely T Cleghorn C Mizdrak A Waterlander W Nghiem N Swinburn B The effect of food taxes and subsidies on population health and health costs: a modelling study. *Lancet Public Health.* (2020) 5:404–13. 10.1016/S2468-2667(20)30116-X 32619542

[23] Caro JC Ng SW Taillie LS Popkin BM. Designing a tax to discourage unhealthy food and beverage purchases: the case of Chile. *Food Policy.* (2017) 71:86–100. 10.1016/j.foodpol.2017.08.001 29375180 PMC5783649

[24] Cobiac LJ Tam K Veerman L Blakely T. Taxes and subsidies for improving diet and population health in Australia: a cost-effectiveness modelling study. *PLoS Med.* (2017) 14:e1002232. 10.1371/journal.pmed.1002232 28196089 PMC5308803

[25] Nnoaham KE Sacks G Rayner M Mytton O Gray A. Modelling income group differences in the health and economic impacts of targeted food taxes and subsidies. *Int J Epidemiol.* (2009) 38:1324–33. 10.1093/ije/dyp214 19483200

[26] Zhen C Finkelstein EA Nonnemaker JM Karns SA Todd JE. Predicting the effects of sugar-sweetened beverage taxes on food and beverage demand in a large demand system. *Am J Agric Econ.* (2014) 96:1–25. 10.1093/ajae/aat049 24839299 PMC4022288

