# Supplementary material for: The Effects of Food Taxes and Subsidies on Promoting Healthier Diets in Iranian Households
Source: Front Nutr. 2022 Jul 13;9:917932. doi: 10.3389/fnut.2022.917932 (PMC9328754; doi:10.3389/fnut.2022.917932)
Supplement: Supplementary file 1 [file Data_Sheet_1.docx]

**Suplementary Appendix**

| Table S1. General characteristics of the participants in Delphi survey | | |
| --- | --- | --- |
| Characteristics | Completed  round 1  (n=27) | Completed  round 1  (n=26) |
| **Gender** | | |
| Male | 18 | 18 |
| Female | 9 | 8 |
| **Job position (n)** | | |
| Nutrition researchers | 6 | 6 |
| Food policy researchers | 3 | 3 |
| Agricultural economists | 10 | 9 |
| Health economists | 4 | 4 |
| Nutrition departments in MoHME | 2 | 2 |
| Non-Communicable Disease  Management Department in MoHME | 1 | 1 |
| NGO | 1 | 1 |
| MoHME, Ministry of Health and Medical Education; NGO, Non-governmental organization. | | |

Tables S2, S3 and S4 shows the Marshallian price elasticities for full sample, low (tertile 1) and high (tertile 3) income households.

| Table S2: Marshallian own- and cross-price elasticities, full sample | | | | | | | | | | | |
| --- | --- | --- | --- | --- | --- | --- | --- | --- | --- | --- | --- |
| sweetened beverages | Legumes | Vegetables | Fruits | Sugar and sweets | Dairies | White meat and eggs | Red meat | hydrogenated oils and animal fats | Un-hydrogenated vegetable oils | Cereals |  |
| -0.01 | 0.02 | -0.04 | -0.02 | 0.02 | 0.05 | -0.04 | 0.06 | 0.01 | 0.08 | **-0.56** | Cereals |
| 0.03 | -0.11 | 0.09 | 0.05 | 0.06 | -0.14 | -0.18 | -0.00 | 0.05 | **-0.93** | 0.06 | Un-hydrogenated vegetable oils |
| -0.02 | -0.03 | -0.05 | -0.05 | -0.02 | -0.06 | 0.07 | -0.1 | **-0.9** | 0.03 | -0.08 | hydrogenated oils and animal fats |
| 0.01 | -0.08 | 0.01 | 0.02 | 0.02 | -0.05 | 0.00 | **-0.88** | -0.09 | -0.01 | 0.01 | Red meat |
| 0.07 | -0.07 | -0.11 | 0.02 | -0.06 | -0.11 | **-0.3** | 0.09 | 0.02 | -0.05 | -0.04 | White meat and eggs |
| 0.06 | -0.01 | 0.03 | 0.12 | 0.05 | **-0.35** | 0.04 | 0.01 | 0.00 | 0.00 | 0.02 | Dairies |
| -0.11 | 0.00 | 0.1 | 0.17 | **-0.74** | 0.16 | 0.06 | 0.06 | -0.09 | 0.08 | 0.19 | Sugar and sweets |
| 0.04 | -0.02 | 0.04 | **-0.6** | -0.03 | -0.06 | -0.17 | -0.07 | -0.02 | -0.14 | -0.05 | Fruits |
| 0.04 | 0.04 | **-0.37** | -0.32 | -0.12 | -0.08 | 0.05 | 0.01 | -0.04 | -0.03 | -0.05 | Vegetables |
| 0.06 | **-1** | -0.07 | 0.16 | -0.06 | 0.03 | -0.05 | -0.1 | 0.02 | 0.02 | 0.06 | Legumes |
| **-0.46** | -0.01 | -0.13 | -0.14 | 0.04 | -0.1 | 0.1 | -0.13 | -0.08 | -0.04 | 0.04 | sweetened beverages |
| Underlined estimates differ significantly from 0 at the 5% significance level. | | | | | | | | | | | |

| Table S3: Marshallian own- and cross-price elasticities, low income households (tertile 1) | | | | | | | | | | | |
| --- | --- | --- | --- | --- | --- | --- | --- | --- | --- | --- | --- |
| sweetened beverages | Legumes | Vegetables | Fruits | Sugar and sweets | Dairies | White meat and eggs | Red meat | hydrogenated oils and animal fats | Un-hydrogenated vegetable oils | Cereals |  |
| -0.01 | 0.04 | -0.06 | -0.03 | 0.03 | 0.09 | 0.05 | 0.06 | 0.03 | 0.1 | **-0.94** | Cereals |
| 0.09 | 0.02 | 0.05 | 0.04 | -0.04 | -0.06 | -0.14 | 0.01 | 0.05 | **-1/1** | 0.11 | Un-hydrogenated vegetable oils |
| -0.09 | -0.14 | -0.1 | -0.08 | -0.08 | -0.12 | -0.03 | -0.1 | **-0.48** | 0.09 | -0.05 | hydrogenated oils and animal fats |
| -0.08 | -0.04 | -0.12 | -0.14 | -0.03 | -0.15 | -0.01 | **-1.07** | -0.07 | -0.15 | -0.19 | Red meat |
| -0.03 | -0.03 | -0.08 | -0.02 | 0.04 | -0.06 | **-0.61** | -0.04 | -0.01 | -0.06 | -0.08 | White meat and eggs |
| -0.03 | -0.06 | 0.12 | 0.12 | -0.08 | **-0.59** | 0.11 | -0.07 | -0.02 | -0.05 | 0.19 | Dairies |
| -0.05 | -0.00 | 0.08 | -0.04 | **-0.95** | -0.05 | -0.00 | -0.01 | 0.01 | 0.02 | 0.04 | Sugar and sweets |
| -0.05 | -0.06 | -0.19 | **-0.67** | -0.07 | -0.28 | -0.13 | -0.1 | -0.07 | 0.04 | -0.24 | Fruits |
| 0.01 | -0.09 | **-0.27** | -0.14 | -0.03 | -0.03 | -0.03 | -0.09 | -0.04 | 0.05 | -0.01 | Vegetables |
| 0.05 | **-0.97** | -0.19 | -0.24 | -0.01 | -0.2 | 0.37 | -0.31 | -0.12 | -0.07 | -0.32 | Legumes |
| **-0.59** | 0.14 | -0.04 | -0.07 | 0.14 | -0.1 | 0.13 | -0.13 | -0.04 | 0.1 | 0.1 | sweetened beverages |
| Underlined estimates differ significantly from 0 at the 5% significance level. | | | | | | | | | | | |

| Table S4: Marshallian own- and cross-price elasticities, high income households (tertile 3) | | | | | | | | | | | |
| --- | --- | --- | --- | --- | --- | --- | --- | --- | --- | --- | --- |
| sweetened beverages | Legumes | Vegetables | Fruits | Sugar and sweets | Dairies | White meat and eggs | Red meat | hydrogenated oils and animal fats | Un-hydrogenated vegetable oils | Cereals |  |
| 0.01 | -0.004 | -0.005 | 0.03 | -0.00 | -0.02 | -0.07 | -0.02 | 0.03 | 0.01 | **-0.76** | Cereals |
| -0.15 | -0.05 | 0.02 | -0.07 | 0.11 | -0.07 | -0.3 | -0.14 | 0.18 | **-1.09** | -0.32 | Un-hydrogenated vegetable oils |
| -0.02 | -0.09 | -0.1 | -0.11 | 0.08 | -0.1 | 0.2 | 0.05 | **-1.06** | 0.47 | -0.25 | hydrogenated oils and animal fats |
| -0.05 | 0.07 | 0.09 | -0.00 | -0.01 | -0.01 | -0.06 | **-0.78** | -0.09 | -0.09 | -0.1 | Red meat |
| 0.00 | 0.08 | -0.00 | -0.04 | 0.00 | -0.1 | **-0.48** | -0.1 | -0.05 | -0.02 | -0.13 | White meat and eggs |
| -0.06 | -0.11 | -0.13 | -0.00 | -0.06 | **-0.43** | 0.09 | -0.1 | 0.04 | -0.07 | -0.04 | Dairies |
| -0.16 | -0.05 | -0.09 | -0.03 | **-0.86** | 0.12 | -0.03 | 0.05 | 0.01 | 0.06 | -0.16 | Sugar and sweets |
| -0.1 | 0.00 | 0.16 | **-1.03** | -0.02 | -0.05 | -0.14 | -0.16 | -0.09 | -0.08 | -0.18 | Fruits |
| 0.01 | 0.07 | **-0.48** | 0.02 | -0.01 | -0.01 | 0.06 | 0.02 | 0.02 | 0.06 | -0.02 | Vegetables |
| -0.09 | **-1.08** | -0.13 | -0.00 | 0.12 | -0.31 | -0.28 | -0.09 | 0.17 | -0.14 | -0.03 | Legumes |
| **-0.44** | 0.02 | -0.07 | -0.08 | -0.08 | -0.1 | -0.09 | 0.05 | -0.09 | 0.02 | 0.00 | sweetened beverages |
| Underlined estimates differ significantly from 0 at the 5% significance level. | | | | | | | | | | | |
